# Physical Characteristics and Biocompatibility of 3D-Printed Polylactic-Co-Glycolic Acid Membranes Used for Guided Bone Regeneration

**DOI:** 10.3390/jfb14050275

**Published:** 2023-05-14

**Authors:** Sidabhat Petposri, Nuttawut Thuaksuban, Supanee Buranadham, Trin Suwanrat, Winita Punyodom, Woraporn Supphaprasitt

**Affiliations:** 1 Department of Oral and Maxillofacial Surgery, Faculty of Dentistry, Prince of Songkla University, Hatyai 90112, Songkhla, Thailand; sidhabhat@gmail.com (S.P.); bomtep.b@gmail.com (T.S.); worapornsup@gmail.com (W.S.); 2 Department of Prosthetic Dentistry, Faculty of Dentistry, Prince of Songkla University, Hatyai 90112, Songkhla, Thailand; supanee.b@psu.ac.th; 3 Department of Chemistry, Faculty of Science, Chiang Mai University, Amphur Muang 50200, Chiang Mai, Thailand; winitacmu@gmail.com

**Keywords:** polylactic-co-glycolic acid, three-dimensional printing, membrane, guided bone regeneration

## Abstract

Bioresorbable polymeric membranes for guided bone regeneration (GBR) were fabricated using the three-dimensional printing technique. Membranes made of polylactic-co-glycolic acid (PLGA), which consist of lactic acid (LA) and glycolic acid in ratios of 10:90 (group A) and 70:30 (group B), were compared. Their physical characteristics including architecture, surface wettability, mechanical properties, and degradability were compared in vitro, and their biocompatibilities were compared in vitro and in vivo. The results demonstrated that the membranes of group B had mechanical strength and could support the proliferation of fibroblasts and osteoblasts significantly better than those of group A (*p* < 0.05). The degradation rate in Group B was significantly lower than that in Group A, but they significantly produced less acidic environment (*p* < 0.05). In vivo, the membranes of group B were compared with the commercially available collagen membranes (group C). The amount of newly formed bone of rat’s calvarial defects covered with the membranes of group C was stable after week 2, whereas that of group B increased over time. At week 8, the new bone volumes in group B were greater than those in group C (*p >* 0.05). In conclusion, the physical and biological properties of the PLGA membrane (LA:GA, 70:30) were suitable for GBR.

## 1. Introduction

Guided bone regeneration (GBR) is the effective procedure for correcting alveolar ridge deficiencies prior to placement of dental implants. The procedure composes placing particulate bone grafts in the defects which serve as an osteoconductive scaffold for regenerating bone and covering barrier membranes for preventing epithelial downgrowth. The membranes should have biocompatibility, the ability for integration with surrounding tissue, adequate mechanical strength for space maintenance or tenting, epithelial cell occlusiveness, good manipulability, and adaptation. Resorbable membranes are most used in routine practice because they are not required removal. Collagen and biodegradable synthetic polymers are commonly used to fabricate resorbable membranes. Collagen membranes exhibit excellent biocompatibility, manageability, and tissue adaptability. The membranes have an average resorption time of 5–28 days and a complete resorption period of 8 weeks, which can prevent soft tissue downgrowth during early wound healing process [1,2,3]. Nevertheless, the degradation periods of the membranes are too short to retain their mechanical strength and space maintenance during the average bone healing process of 3 months or longer if xenogeneic bone substitutes are used [4]. Therefore, collagen membrane usage is limited for GBR in small intra-bony defects and is unsuitable for large amounts of bone augmentation. For the synthetic polymers, polylactic acid (PLA), polyglycolic acid (PGA), polylactic-co-glycolic acid (PLGA), and polycaprolactone (PCL) have been commonly used to fabricate resorbable membranes [5,6]. The major advantage of polymeric membranes over conventional collagen membranes is that various safe non-xenogeneic or medical-grade materials can be used either singly or in combination. Therefore, the physical and biological properties of polymeric membranes can be easily altered and controlled. In recent years, PLA and PCL have been extensively investigated due to their biocompatibility, processability, low cost, and excellent mechanical strength. Nevertheless, the average resorption periods of PLA and PCL in vivo are 1–2 years and 2–3 years, respectively, which is too long for them to be used as membranes for GBR [7,8]. PLGA is composed of lactic acid (LA) and glycolic acid, (GA) and ratios of these copolymers play an important role in determining its degradability [1]. Theoretically, PGA is more hydrophilic than PLA; therefore, the degradation rate of PLGA can be increased or decreased by increasing the proportions of GA or LA, respectively [8,9]. PLGA has been used as a raw material for developing various resorbable medical devices, including sutures, scaffolds, membranes, and controlled-release drug delivery carriers, owing to its biocompatibility and controllable biodegradability [10]. Medical devices made of PLGA with various LA:GA ratios are commercially available. The absorbable suture (Vicryl^®^), made from PLGA (10% L-lactide and 90% glycolide), can maintain functional strength over 28 days and shows complete resorption within 4 months. The commercial PLGA membranes have functional times of 8–10 weeks, with complete resorption durations ranging from 5 to 24 months [10,11,12,13,14,15,16]. For instance, the Cytoflex Resorb^®^ membrane has a barrier function of over 2 months and is completely absorbed within 6 months. The Resolut^®^ membranes have a functional time of 8–10 weeks and a complete resorption period of 5–6 months. Takata et al. compared the effects of eight commercial GBR membranes on the migration of osteoblasts that were seeded on the membranes in vitro [17]. The experiment groups included bovine type I collagen, porcine type I collagen, bovine type I atelocollagen, PLA, PLGA, and expanded polytetrafluoroethylene (e-PTFE) membranes. The results demonstrated that the PLGA membranes had the greatest cell migration, whereas the collagen and e-PTFE membranes had the lowest cell migration. According to the authors, osteoblasts have a good affinity for the glycolide polymer of the PLGA membranes; therefore, the cells could attach well to the fibers of the membranes. Wadhawan et al. compared the effects of non-resorbable e-PTFE and bioabsorbable PLGA membranes when combined with bioactive glass bone substitutes for GBR of periodontal intra-bony defects [18]. The results demonstrated that both types of membrane groups could significantly reduce the probing depth and increase periodontal defect fill over a clinical observation period of 9 months. However, no study has compared the physical and biological properties of PLGA membranes with different LA:GA ratios fabricated using the same technique.

Our center has developed polymeric scaffolds using various in-house filament-based techniques [19,20,21,22]. Filaments composed of PCL and PLGA with an average diameter of 1.7 ± 0.05 mm have been adapted to a fused deposition modeling technique for fabricating the scaffolds [23] and membranes using a 3D printer, respectively.

In this study, the physical characteristics and biocompatibility of the PLGA membranes with high LA–low GA and low LA–high GA ratios were compared to determine the optimal ratio for the GBR procedure.

## 2. Materials and Methods

### 2.1. Study Groups

In group A, membranes were fabricated from PLGA (LA:GA = 10:90), and in group B from PLGA (LA:GA = 70:30).

### 2.2. Fabrication of the Biodegradable Membranes

**Figure 1:**
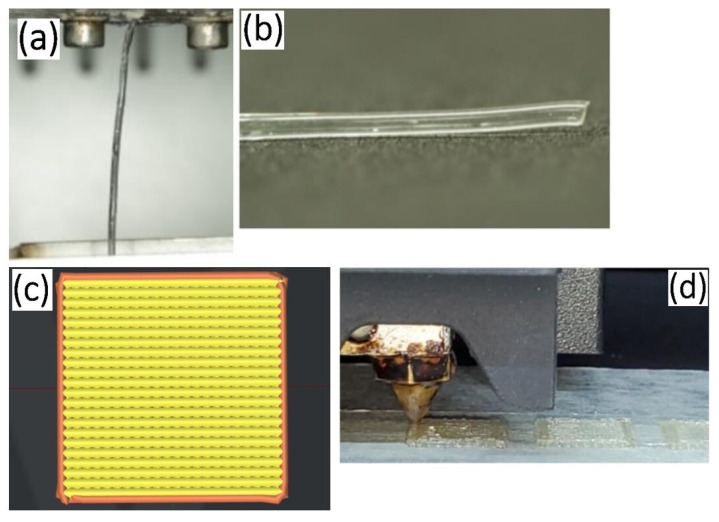
(**a**) Extruding process of the PLGA filament. (**b**) Stocked PLGA filament. (**c**) Preview design of the membrane. (**d**) Printing process of the membranes.

Medical-grade PLGAs, including PLGA (LA:GA = 10:90 mol%) (Purasorb PLG 1017, Corbion, The Netherlands) and PLGA (LA:GA = 70:30 mol%) (CMU-Bioplasorb PLG, Chiang Mai, Thailand), were used as raw materials for groups A and B, respectively. Firstly, the materials for both groups were melted in the chambers of a melting-extruding machine at 150–210 °C and then extruded through the 1.0 mm nozzle tip to form the filaments (Figure 1a) [23]. The filaments were stocked for 3D printing (Figure 1b). The architecture of the membrane was designed using 3D Slicer Software (ideaMaker version 4.1.0.4990, Raise-3D Technologies Inc., Irvine, CA, USA) in a grid infill pattern at 0° and 90° of filament rows, and the layer height was set to 0.05 mm with the infill density of 90% for printing eight layers per membrane (Figure 1c). The filaments were extruded through a 0.6 mm nozzle tip of the 3D printer (RAISE3D-E2, Raise-3D Technologies Inc., Irvine, CA, USA) at an extruder temperature of 210 °C for group A and 180 °C for group B (Figure 1d). The membranes were sterilized using ethylene oxide gas (ethylene oxide 100%, 37 °C, humidity 76%, 2 h) and kept in dry condition for at least 2 weeks before tests.

### 2.3. Morphologies and Microstructures

The surface morphologies of the 10 mm × 10 mm membranes were assessed using a stereomicroscope (Nikon SMZ1500, Nikon, Tokyo, Japan) and scanning electron microscope (SEM, JOEL Ltd., Tokyo, Japan) (*n* = 3).

### 2.4. Mechanical Properties

The 50 mm × 10 mm membrane specimens were immersed in simulated body fluid (SBF) at room temperature for 3 h prior to the tests. The SBF was prepared in accordance with Kokubo et al. [24]. In the wet stage, each specimen was secured at an initial distance of 30 mm between the grips of a universal machine (Lloyd Instruments Ltd., West Sussex, UK) (*n* = 5/group). A tensile force was applied at a speed of 3 mm/min until the specimen broke. The mechanical properties of the membranes were analyzed using an analysis software (NEXYGEN, Lloyd Instruments Ltd., Hampshire, UK).

### 2.5. Surface Wettability

Surface wettabilities of the membranes were analyzed using an optical contact angle analyzer (OCA25, DataPhysics Instruments GmbH, Filderstadt, Germany). The sessile drop method was used to measure the water contact angles (WCA) by depositing 5 µL deionized water on the surfaces of the 10 mm × 10 mm membranes (*n* = 3/surface/group).

### 2.6. Degradability

The membranes were immersed in SBF to assess their weight loss (%) and pH change (*n* = 5/group/time point). The pH change from the baseline pH of the SBF was measured using the pH meter (SevenCompact, Mettler Toledo GmbH, Greifensee, Switzerland). Then, the 10 mm × 10 mm membrane specimens were immersed in 4 mL of SBF/tube in 50 mL centrifuge tubes (Corning, Merck KgaA, Darmstadt, Germany). The tubes were incubated at 37 °C, and the pH of the solution of each membrane was measured on days 1, 3, 7, 14, and 21. The control group consisted of a solution without membranes. To measure the weight loss, each membrane was weighed (Wd0) using an analytical balance (Satorius, Goettingen, Germany) before immersion in 4 mL of SBF/well in 12-well plates (Corning, Merck KgaA, Darmstadt, Germany). The membranes were incubated at 37 °C, and the SBF was added every 10 days to maintain a constant volume. To measure the weight loss on days 15, 30, 60, 90, and 120, the membranes were collected, rinsed with distilled water, and freeze-dried in a freeze dryer (LaboGene, Lillerød, Denmark) for 3 h. Their dry weights (Wdt) were measured, and their weight loss was calculated as:

%Weight loss = 100 × (Wd0 − Wdt)/Wd0

The morphology of the membranes during the test was evaluated via SEM (*n* = 2/time point).

### 2.7. Biocompatibility

#### 2.7.1. Cell Proliferation 

Mouse osteoblast cell line (MC3T3-E1, ATCC, Manassas, VA, USA) and mouse fibroblast cell line (L929, ATCC, Manassas, VA, USA) were grown in the proliferation medium [α-Modified Eagle Medium medium (Gibco, Thermo Fisher Scientific, Waltham, MA, USA) supplemented with 10% fetal bovine serum (Gibco, Thermo Fisher Scientific Inc., Waltham, MA, USA)] until passages 3 to 5. Prior to cell seeding, 10 mm × 10 mm membranes were immersed in the proliferation medium for 24 h. Afterwards, the cells were seeded on the membranes of both groups at a density of 2 × 10^4^ cells/cell type/membrane (*n* = 5/cell type/group/time point). The plates were cultivated in 5% CO_2_ at 37 °C and amounts of the cells in the cell-membrane construct of each well were measured on days 1, 7, and 14 using the PrestoBlue reagent (Thermo Fisher Scientific Inc., Waltham, MA, USA) at the optical density of 600 nm.

#### 2.7.2. SEM

On days 1, 7, and 14, after seeding, the constructs were fixed in 2.5% glutaraldehyde (Sigma-Aldrich Inc., St. Louis, MO, USA) for 2 h. The specimens were dehydrated in a 50–100% ethanol series and coated with gold–palladium. The characteristics of the cells in the constructs were observed via SEM (*n* = 2/cell type/group/time point).

#### 2.7.3. Biocompatibility in Rat Models

The results of the degradation test showed that the membranes of group A lost their integrity on day 14. Therefore, only the membranes of group B were used in the experiment and compared with the commercially available resorbable collagen membranes. The experiment in twenty-four male Wistar rats (Nomura Siam International, Bangkok, Thailand) was approved by Institutional Animal Care and Use Committee, Prince of Songkla University (MHESI 68014/1598). After general anesthesia by the intraperitoneal administration of ketamine (60 mg/kg) and xylazine (10 mg/kg), a bicortical calvarial defect (8 mm in diameter) was created in each animal. In group B, the defect was covered with a membrane. In group C, the defect was covered with a collagen membrane (OssGuide, non-crosslinked collagen membrane, SK bioland Co., Ltd., Seoul, Republic of Korea). Wounds were closed using 4/0 absorbable sutures (Vicryl, Ethicon LLC, London, UK). All animals received subcutaneous cephalexin (10 mg/kg) and buprenorphine 0.1 mg/kg once daily for 3 days for postoperative antibiotic prophylaxis and analgesia, respectively. At 2, 4, and 8 weeks postoperatively, the animals were sacrificed by the intraperitoneal administration of an overdose of 120 mg/kg of pentobarbital sodium. Calvarial specimens at the areas of bone defects were collected and fixed in 10% formalin.

#### 2.7.4. Microcomputed Tomography (µ-CT) Analysis

The specimens were coronally scanned using a µ-CT machine (µ-CT 35, SCANCO Medical AG, Wangen-Brüttisellen, Switzerland) (*n* = 4/group/time point). New bone formation within each defect site was measured as the new bone volume fraction (VF) using an analysis software (µ-CT 35 Version 4.1, SCANCO Medical AG, Wangen-Brüttisellen, Switzerland) with the following formula:

New bone VF = [New bone volume ÷ Total defect volume] × 100

#### 2.7.5. Histological Assessment

The specimens were decalcified in 14% ethylenediaminetetraacetic acid (EDTA) and embedded in paraffin (*n* = 4/group/time point). Serial sections were cut at positions 500 µm from the midline of each specimen and stained with hematoxylin and eosin (H&E) (2 sections/specimen). Microscopic features of the stained section were assessed descriptively.

### 2.8. Statistical Analysis

Data were analyzed using SPSS version 14 (IBM Corporation, Armonk, NY, USA). The morphologies of the membranes and microscopic features of the cells-membrane constructs were assessed. An independent *t*-test and one-way analysis of variance (ANOVA) followed by Tukey’s HSD were applied to compare the differences in the quantitative parameters among the experimental groups and time points at *p* < 0.05.

## 3. Results

### 3.1. Morphologies and Microstructures

**Figure 2:**
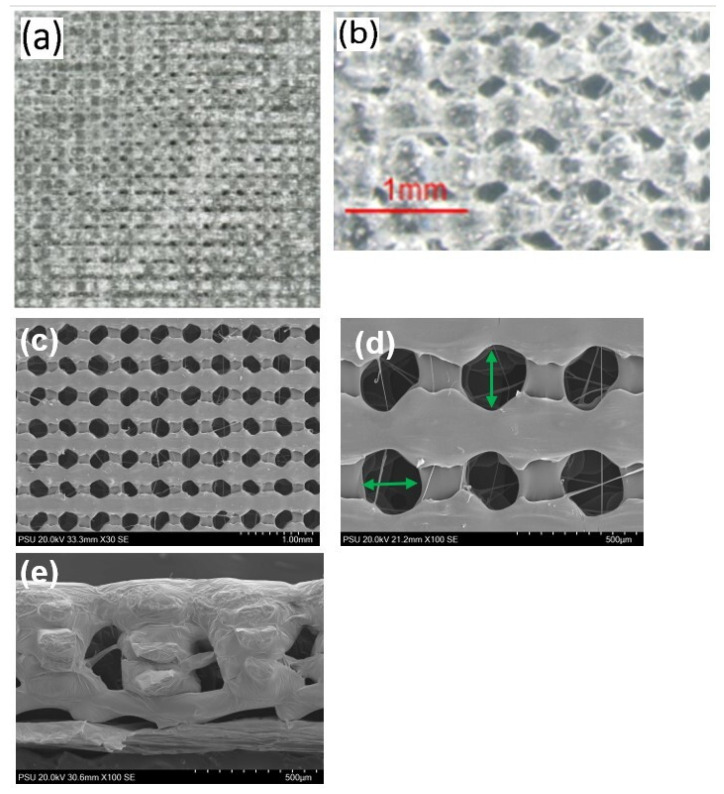
(**a**) Stereomicroscopic image showing the architectures of the membrane. (**b**) The image at a magnification of 50× showing the morphologies of the membrane surface. (**c**) SEM image (30×) showing regular porosity of the membrane. (**d**) SEM image (100×); the green arrows indicate the lines for measuring the pores sizes. (**e**) SEM (100×) image showing the porosity of the lateral aspect of the membrane.

The microscopic features of the membranes are shown in Figure 2. The membranes had the regular porosity with average pore size and thickness of 232 ± 23 µm and 0.65 ± 0.02 mm, respectively.

### 3.2. Mechanical Properties

The mechanical properties of the membranes are shown in Table 1. The tensile strength and Young’s modulus of group B were significantly higher than those of group A, whereas the percent strain at the maximum load of group A was significantly higher than that of group B (*p* < 0.05).

### 3.3. Surface Wettability

WCA of the membranes of group A and B were 102.59 ± 1.13 and 101.71 ± 0.38, respectively, with no statistical difference (*p* > 0.05).

### 3.4. Degradability 

**Figure 3:**
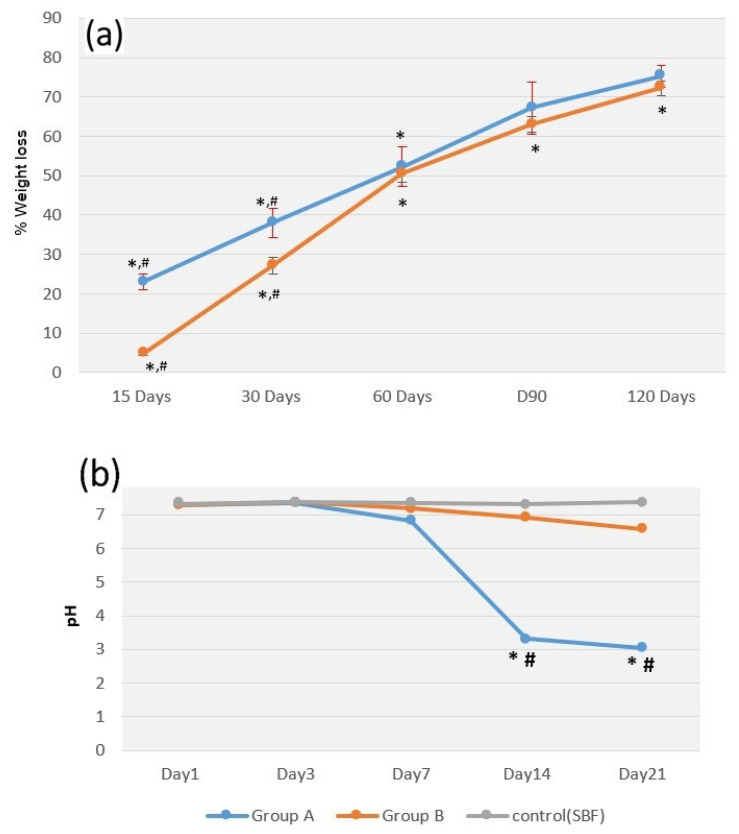
(**a**) Graph of the degradation of PLGA membranes over 120 days. (**b**) Graph of pH changes of SBF over 21 days. ^#^ *p* < 0.05: comparing between the groups at each time point; * *p* < 0.05: comparing among the time points within each group.

**Figure 4:**
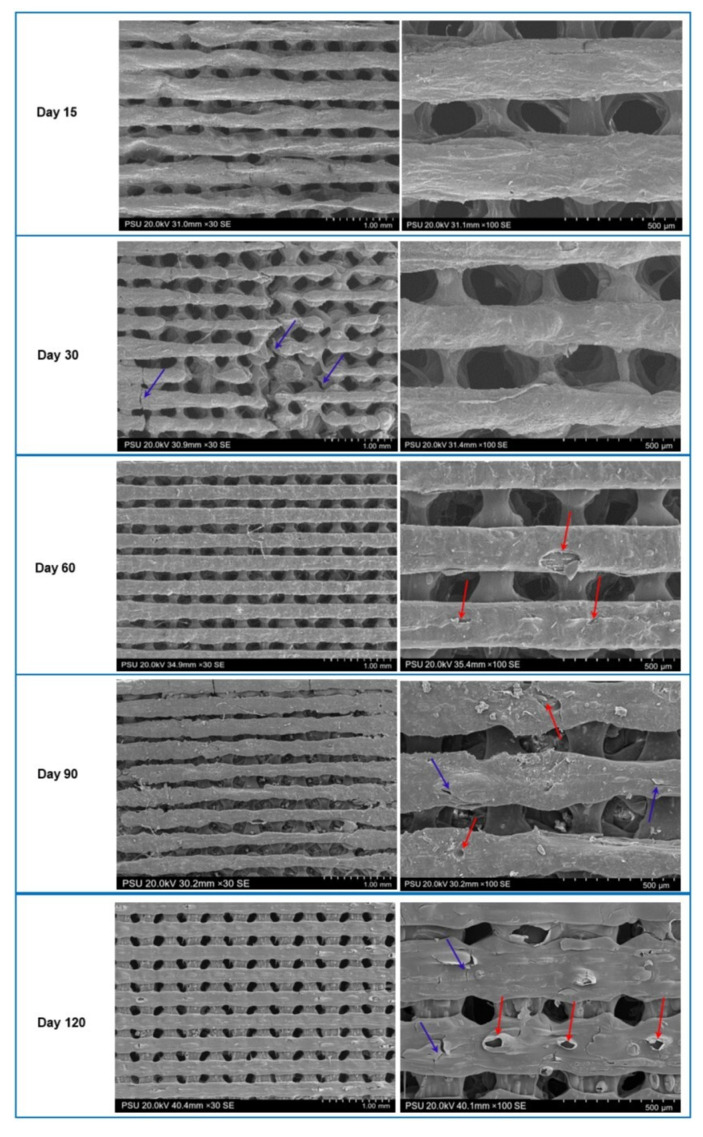
SEM images of the membranes of group B over the period of the degradation test of 120 days. The red arrows indicate the erosive areas, and purple arrows indicate the crack lines.

Membrane degradation profiles are shown in Figure 3a. The membranes significantly degraded with time over 120 days (*p* < 0.05), and average percentages of weight loss in groups A and B were 75.26 ± 2.89 and 72.24 ± 1.82%, respectively. In the first 30 days, degradation in group B was significantly lower than that in group A (*p* < 0.05). The change in the pH of the SBF containing the membranes is shown in Figure 3b. The pH of group B specimens slightly decreased over 21 days, whereas that of group A specimens gradually decreased during the first 7 days and then significantly decreased thereafter (*p* < 0.05). On days 14 and 21, the levels of pH in group A were significantly lower than those in group B (*p* < 0.05). Notably, the membranes of group A could not be processed by SEM because of their loss of integrity from day 15 on. SEM images of the membranes of group B at the observation time points are shown in Figure 4. Erosive areas and crack lines on the membrane surfaces were observed on day 30.

### 3.5. Cell Proliferation

**Figure 5:**
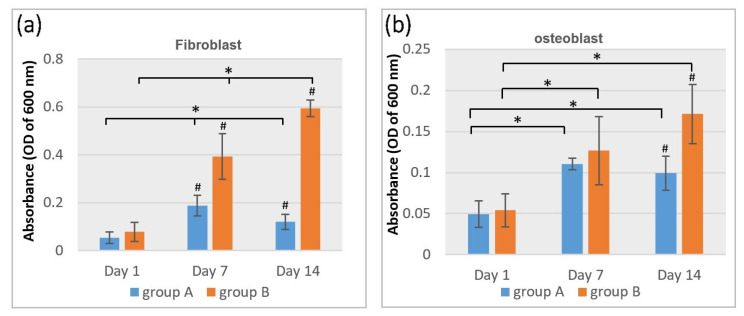
Bar graphs of cell proliferation on PLGA membranes, (**a**) fibroblasts and (**b**) osteoblasts. ^#^ *p* < 0.05; comparing between groups, * *p* < 0.05; comparing among the time points within a group.

Figure 5 shows the cell proliferation on days 1, 7, and 14. For both cell types, the OD of group B increased remarkably with time, whereas the number of cells in group A increased in the first 7 days but decreased thereafter. For fibroblasts, the OD of group B was significantly higher than that of group A on days 7 and 14 (*p* < 0.05). For osteoblasts, no statistically significant difference was observed between the ODs of both groups during the first 7 days. On day 14, the OD of group B was significantly higher than that of group A (*p* < 0.05).

### 3.6. Microscopic Observation

**Figure 6:**
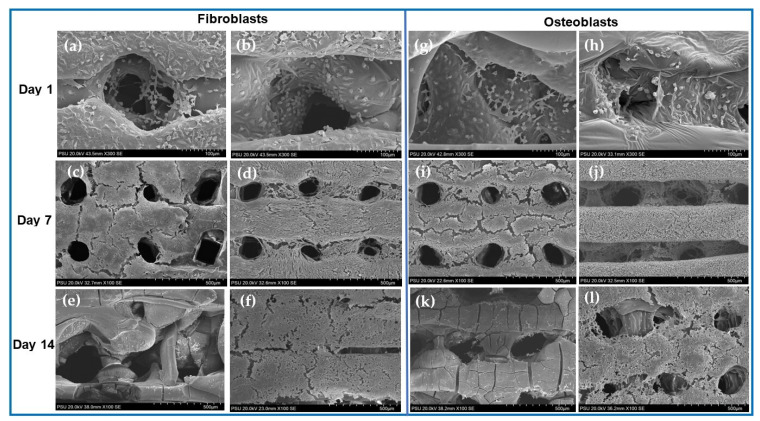
SEM images of cells proliferated on PLGA membranes in group A: fibroblasts (**a**,**c**,**e**) and osteoblasts (**g**,**i**,**k**) and group B: fibroblasts (**b**,**d**,**f**) and osteoblasts (**h**,**j**,**l**). On day 1, the cells attached well on the surfaces of the membranes in both groups. On day 7, the multi-layers of the cells covered almost all the surfaces of the membranes in both groups. On day 14, the cells on the membranes in groups A decreased remarkably and detached from the membrane surfaces, whereas those on the membranes in group B continued growing.

The fibroblasts and the osteoblasts could attach and grow well on the surfaces of the membranes in both groups on the first 7 days. On day 14, the cells of group B continued to grow in multiple layers, whereas the cells of group A remarkably decreased in number and detached from the surfaces of the membranes (Figure 6).

### 3.7. Experiment in Rat Models

The animals showed good tolerance to surgery. All surgical wounds healed spontaneously without dehiscence or infection.

#### 3.7.1. µ-CT Analysis

**Figure 7:**
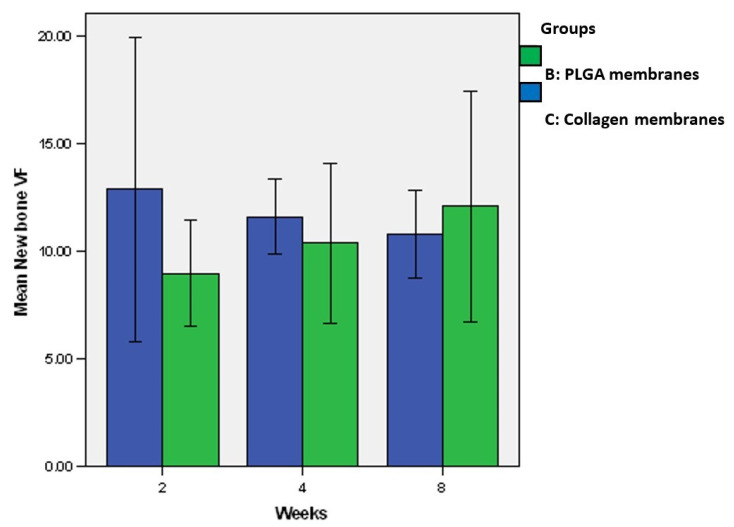
Profiles of the new bone VF of groups B and C. During the first two weeks, the newly formed bone in group B was slightly less developed than that in group C. At week 8, the new bone VF in group B were greater than those in group C. No significant difference was detected.

**Figure 8:**
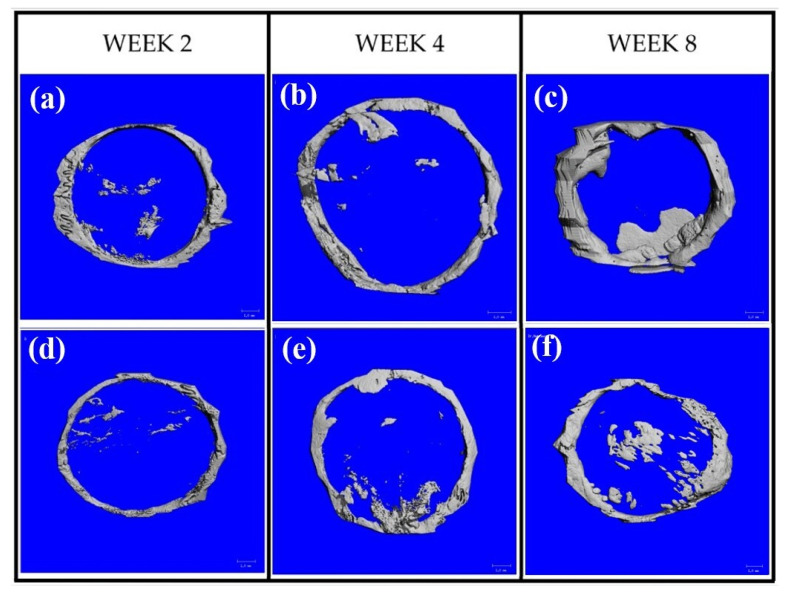
New bone regeneration within the defect sites of group B: (**a**–**c**) and group C: (**d**–**f**). In both groups, most of newly formed bone regenerated from the peripheries of the defects and increased with time. Some new bone areas were in the middle portions.

Figure 7 shows the new bone VF of the defects. At weeks 2 and 4, the amount of newly formed bone in group B was lower than that in group C (*p* > 0.05). At week 8, the new bone volumes in group B were greater than those in group C (*p* > 0.05). The new bone VF in group C appeared to be stable after week two, whereas that in group B increased with time. Figure 8 shows the constructed images of the bone defect sites. From week 2, newly formed bone regenerated mainly at the peripheries of the defects in both groups. However, some areas of bone formation were found in the middle portions.

#### 3.7.2. Histological Assessment

**Figure 9:**
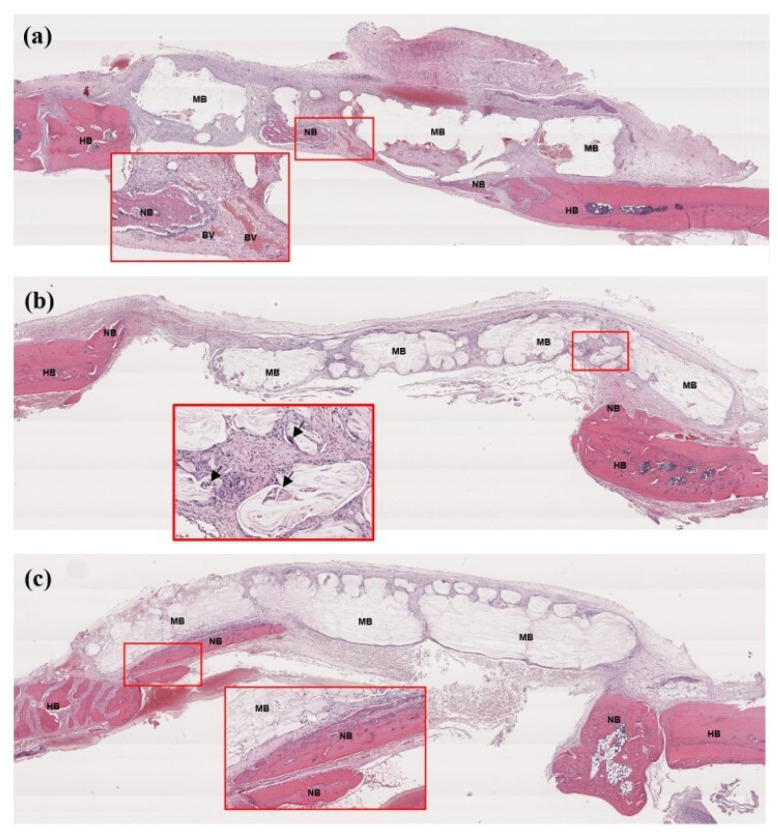
Histological features of the bone defects in group B; (**a**): week 2, (**b**): week 4, (**c**): week 8. At week 2, the PLGA membrane was surrounded by dense fibrous tissue. Areas of newly formed bone formation were detected at the peripheries and in the middle of the defect (see box). At week 4, the new bone continued regenerating from the peripheries, and giant cells were occasionally found ingesting particles of the PLGA membrane (see box and arrows). At week 8, the large new bone was found regenerating along the inferior aspect of the membrane (see box). HB = host bone, NB = new bone, MB = the PLGA membrane, BV = blood vessel.

**Figure 10:**
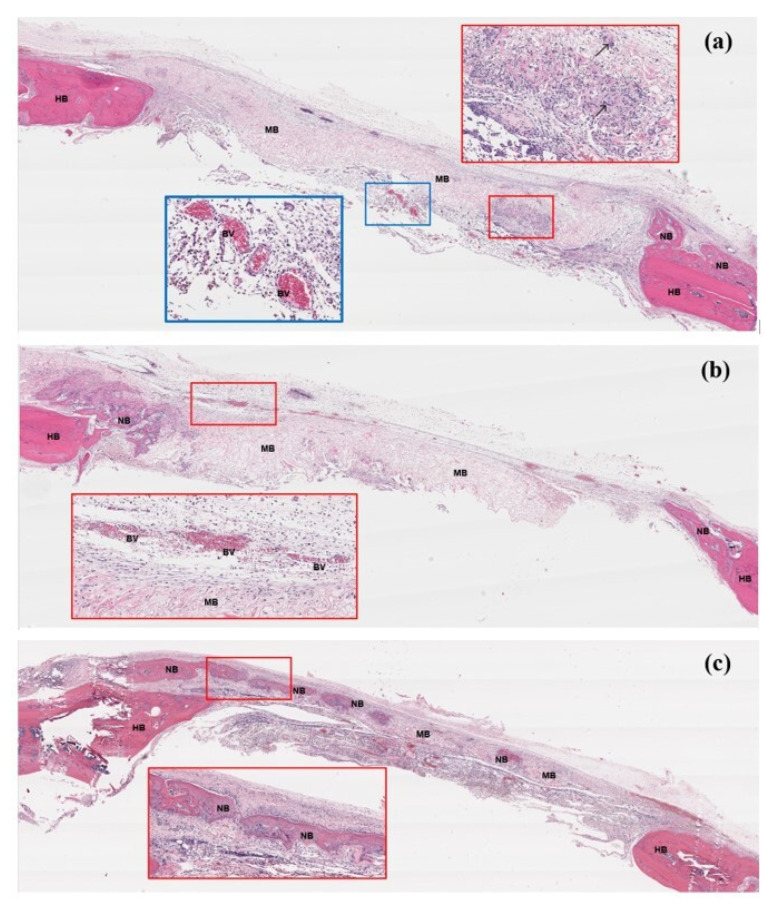
Histological features of the bone defects in group C; (**a**): week 2, (**b**): week 4, (**c**): week 8. At week 2, dense fibrous tissue and chronic inflammatory cells (see red box) including giant cells (arrows) were detected surrounding the collagen membrane. Newly formed bone was detected at the peripheries of the host bone. New blood vessels were detected in some areas beneath the membrane (see blue box). At week 4, the new bone was found regenerating from the peripheries into the middle portion of the defect. The membrane was still detected. Larger blood vessels were formed along the superior aspect of the membrane (see box). At week 8, the larger new bone regenerated along the area of the membrane (see box). Remnants of the membranes were still detected in some areas. HB = host bone, NB = new bone, MB = the collagen membrane, BV = blood vessel.

Figure 9 and Figure 10 show the histological features in groups B and C, respectively. The PLGA membranes were observed as empty spaces because they were dissolved by histological processes. From week 2, newly formed bone was detected in both groups, regenerating from the periphery of the host bone. In group B, the new bone was observed in the middle of the defects. During the observation period, the membrane remnants were detected in both groups. The collagen membranes seemed to have collapsed into the defect, whereas the PLGA membranes were slightly swollen but could cover the defects without collapsing. At week 8, a larger new bone was detected, regenerating along the area of the collagen membrane remnant and the inferior aspect of the PLGA membrane.

## 4. Discussion

In recent years, 3D printing has emerged as a popular technique for fabricating various types of biomedical devices, including scaffolds and membranes. The architectures of the devices, including pore size, porosity, and thickness, were designed using computerized design software that can be easily customized for different purposes prior to fabrication. In this study, 3D-printed membranes were fabricated from stocked PLGA filaments. Using a custom-made melting–extrusion machine, both ratios of PLGA could be processed as filaments without any cytotoxic solvents. The PLGA materials used in this study were of medical grade that were appropriate for fabricating membranes for clinical practices. The PLGA (LA:GA = 70:30) synthesized in our collaboration laboratory is remarkably cheaper than imported PLGA and can significantly reduce the cost of the membrane fabrication. The ratios of LA:GA in the PLGAs, 70:30 and 10:90, used for fabricating the membranes were similar to the most commonly used ratios in commercial PLGA membranes and sutures of LA:GA = 85:15 (Resolut) and 10:90 (Vicryl), respectively. In a previous study, Kim et al. fabricated 3D-printed membranes made of PLGA (PLA:PGA = 10:90%, Purasorb 1017), which is the same material used in our study [25]. The membranes were implanted subcutaneously in Chinchilla rabbits, and their histological results at 28 days were compared with the corresponding results of pure PLA membranes. The authors found that PLGA membranes produced fewer inflammatory and foreign-body reactions. The biodegradation of these membranes began 1–2 weeks after implantation and increased with time until the final stage of hydrolytic degradation on day 28. The authors concluded that PLGA (10:90%) appears to be suitable for fabricating GBR membranes in terms of function and complete resorption periods. A previous histological study demonstrated that the Resolut membrane (LA:GA = 85:15) retains its structure for 4 months and is resorbed completely within 5–6 months [26,27]. We expect that the complete degradation of the PLGA membranes should occur within 4 months from the start of the dental implant placement. Therefore, PLGA with higher GA (LA:GA = 70:30) was selected for this study, and it was hypothesized to increase the degradation rate of the membranes. Our membranes were found to have better mechanical strength than some commercially available collagen membranes that were tested in similar conditions, such as BioGide membranes (1.68 ± 0.54 MPa) and Ossix Plus (1.2 ± 0.14 MPa), and their Young’s moduli were within a range of 30–700 MPa of the commercially available bioresorbable membranes [28,29,30,31]. In this study, the osteoblasts and fibroblasts, which play an important role in the mechanism of GBR, were tested. Regarding surface wettability, the membranes of both groups had high contact angles, implying hydrophobic behavior of their surfaces. However, both cells could attach and grow well on the surfaces of membranes. It is presumed that the surface properties of the membranes were suitable for the attachment of the fibroblasts and the osteoblasts. For the degradation behavior, the ester linkages of PLGA were hydrolysed in an aqueous environment to form lactic and glycolic acids, which were finally broken down to be carbon dioxide and water [5]. The results revealed that the membranes of both groups degraded by more than 50% and 70% within 60 and 90 days, respectively. This seemed to be commensurate with the bone healing process [32]. The degradation rates of PLGA depend on the different composition ratios of LA and GA. Initially, the PLGA (10:90) membrane was expected to be rapidly resorbable, comparable to the collagen membrane, and the PLGA (70:30) membrane was expected to be slowly resorbable. Nevertheless, the higher composition of the hydrophilic PGA would affect the degradation behaviours of the PLGA. The PLGA (10:90) membranes had too rapid degradation during the first 30 days (significantly higher than the PLGA (70:30) membranes), and the membranes lost their stability on day 15. In addition, they produced a more acidic environment owing to the degradation of the lactide and glycolide copolymers after day 7, which was unsuitable for cell viability. These conditions corresponded to the proliferation profiles of both cell types. The viability of the cells cultured on the PLGA (10:90) membranes decreased after day 7, whereas the cells grew well on the PLGA (70:30) membranes until day 14. Therefore, from our results, PLGA (LA:GA = 90:10) is not a suitable raw material for fabricating GBR membranes. In the animal models, only PLGA (70:30) membranes were tested, and the results were compared with those of commercially available collagen membranes. The amount of newly formed bone in the defects covered with collagen membranes was stable after week 2, whereas that in the defects covered with PLGA membranes increased with time. In the human body, the degradation mechanisms of collagen and PLGA membranes are different. The collagen membrane is mainly degraded by the enzymatic digestion of macrophages and polymorphonuclear leukocytes, whereas the PLGA membrane is degraded via hydrolysis or macrophage cell response [25,33]. Therefore, the presence of chronic inflammatory cells and giant cells was associated with the degradation kinetics of both membrane types during the first two weeks. In addition, the PLGA membranes appeared slightly swollen, indicating water uptake during hydrolysis. Notably, the two types of membranes were still detectable after eight weeks. However, the collagen membranes collapsed into the defect spaces, whereas the PLGA membranes retained their shape and maintained the space beneath the membranes over the experiment period. The new bone was mainly regenerated from the peripheries of the host bone; however, most areas of new bone and new blood vessels were detected along the bottom surfaces of the membranes. This implies that the membranes can act as osteoconductive platforms for new bone and vascular regeneration. The pore sizes and interconnecting structures of membranes are very important factors for transporting essential proteins and growth factors, migrating progenitor cells, and regenerating new blood vessels, which are important for new bone formation. In addition, these factors tighten the integration of the membranes and adjacent tissues, which can reduce the separation and exposure of the membranes [34]. However, the optimal pore size that can simultaneously prevent soft tissue ingrowth and promote new vessel and bone regeneration has not been determined [35,36]. Several studies have shown that macroporous membranes can facilitate higher rates and quantities of bone regeneration than microporous membranes [37,38,39]. Lundgren et al. reported that membranes with pore sizes of 25–300 µm would be optimal for underneath new bone regeneration [40]. In addition, a totally occlusive barrier membrane delays the rate of bone tissue augmentation owing to inadequate penetration of capillaries, which tend to be filled with avascular tissues. Correspondingly, some studies supported the idea that the pore size of 100 μm is the smallest size for migration of osteoprogenitor cells and enhancement of angiogenesis [34,41,42] and that the pore size should be greater than 150 µm for new bone formation [37]. In this study, the membranes were designed to be macroporous with an average pore size of 232 ± 23 µm on both sides, which was hypothesized to allow for vessel and osteoprogenitor cell regeneration and increase the integration with surrounding tissue, which enhances membrane stability. However, a larger pore size allows fibroblasts to overpopulate the bone defect area, which inhibits the growth and function of osteoblasts [39]. This would explain why less bone formation was detected in the PLGA membrane group during the first four weeks in the animal models than in the collagen membrane group. Unlike the collagen membrane, the PLGA membrane can maintain the defect space by itself; therefore, the membrane is probably used solely for GBR without being supported by the underlying bone substitutes. In principle, bioactive ceramic materials can be easily added to polymeric membranes using melt blending techniques. Shim et al. fabricated PCL/PLGA (LA:GA = 85:15)/β-tricalcium phosphate (β-TCP) membranes using solid freeform fabrication technique at the ratio of PCL:PLGA:β-TCP = 40:40:20 by weight percentage and implanted the membranes in rabbits’ calvarial defects [43]. The results showed that amount of newly formed bone in the PCL/PLGA/β-TCP membrane group were significantly greater than that in the PCL/PLGA membrane group after week 4 and the bone could bridge the defects within 8 weeks without additional bone substitutes. Therefore, to improve the bioactivity of our prototype PLGA membranes, the addition of some bioactive ceramic materials, such as BCP and β-TCP, should be adapted for the 3D printing technology.

## 5. Conclusions

PLGA membranes were successfully fabricated using 3D printing technology. Membranes made of PLGA (LA:GA = 70:30) were suitable for GBR owing to their good mechanical properties, degradability, and biocompatibility.

## Figures and Tables

**Table 1:** Summary of the mechanical properties of the membranes. (* *p* < 0.05).

Groups	Tensile Strength (MPa)	Strain at Maximum Load (%)	Young’s Modulus (MPa)
A	1.64 ± 0.29	10.45 ± 1.06	40.50 ± 6.66
B	2.12 ± 0.62	2.49 ± 0.73	99.92 ± 12.36
*p*-value	0.049 *	0.000 *	0.000 *

## Data Availability

The data presented in this study are available on request from the corresponding author.
